# A Descriptive Analysis of Unique Disability Identification Card (UDID)-Certified Visually Disabled Patients at a Tertiary Eye Care Center in Central India

**DOI:** 10.7759/cureus.31106

**Published:** 2022-11-04

**Authors:** Kavita A Dhabarde, Arti B Wankhade, Pallavi M Doble, Nisha V Rahul, Rohit P Kende

**Affiliations:** 1 Department of Ophthalmology, Government Medical College and Hospital, Nagpur, IND

**Keywords:** visual rehabilitation, government programs, low vision aids, retinitis pigmentosa, disability certificate, blindness, visual impairment, visual disability, udid

## Abstract

Objective

In this study, we aimed to examine the demographic characteristics, causes, and severity of visual disability and the reasons for seeking disability certificates among Unique Disability Identification Card (UDID)-certified visually disabled patients at a tertiary eye care center in central India.

Materials and methods

A retrospective observational analysis of medical records and data from the UDID portal involving 600 visually disabled individuals who were certified between February 2019 to March 2022 was performed. Demographic characteristics, diagnosis of the ocular disease, primary etiology, and percentage and grade of visual disability, as well as the main reasons for seeking a visual disability certificate, were analyzed statistically. Best-corrected visual acuity of less than 6/24 to 3/60 or a visual field less than 40 degrees to 10 degrees around the center of fixation or hemianopia involving the macula in the better eye were included in the low-vision category. Best corrected visual acuity of less than 3/60 to "no light perception" or visual field less than 10 degrees around the center of fixation in the better eye were included in the blindness category.

Results

Out of the total 600 patients, 214 (35.67%) were in the age group of 11-30 years. There were more males (63.67%) than females (36.33%) in the study. Four hundred patients (66.67%) had 100% disability. Retinal diseases (n=229, 48.50%) including retinitis pigmentosa (RP) (n=130, 21.67%) were the most common cause of visual disability. Travel concessions and Government allowances were the most common reasons for seeking a disability certificate.

Conclusion

The study highlights the leading causes of visual disability, and RP was found to be the most common one. Avoidance of consanguineous marriages and genetic counseling should be made mandatory to prevent blindness due to RP. We recommend the widespread institution of eye care facilities, increasing the availability of healthcare facilities to all, and community education to eliminate avoidable blindness. This study provides key data to the Government to implement new policies or change the existing ones, plan for future strategies, and prioritize the rehabilitation of visually disabled individuals. Government programs to increase awareness among unregistered visually disabled patients regarding the benefits and rehabilitative measures like UDID card and low vision aids is the need of the hour.

## Introduction

Blindness is the most catastrophic reality in the lives of the visually handicapped. Blindness has a major impact on the emotional, personal, physical, educational, social, and economic well-being of individuals. In India, the Ministry of Social Justice and Empowerment (MoSJE) is responsible for developing programs to support persons with disabilities including visual impairment. Globally, at least 2.2 billion people have near or distant vision impairment [1]. In India, currently, there are an estimated 4.95 million blind persons and 70 million visually impaired individuals, out of which 0.24 million are blind children [2]. In almost half of these cases, vision impairment could have been prevented or has yet to be addressed. Providing visually disabled individuals a ray of hope to live their life happily, reducing day-to-day problems they face, instilling in them a positive attitude to lead fulfilling lives, and rehabilitating the blind in terms of social, educational, vocational, and financial support is the need of the hour.

The Unique Disability Identification (UDID) project serves as a tool to enhance and enrich the life of the visually disabled by not only providing them a certification of their blindness but also playing a pivotal role in their rehabilitation. The UDID card brings with it a host of benefits to persons with disabilities so that they do not need to make multiple copies of documents or maintain multiple documents as the card captures all the necessary details, which can be decoded with the help of a reader. UDID card enables the tracking of the physical and financial progress of beneficiaries at all levels of the hierarchy of implementation: the village, block, district, state, and national levels [3].

The UDID project was instituted and organized by MoSJE in India in 2016. At our institute, which is a tertiary eye care hospital in Maharashtra in central India, the project was started in June 2018. Since the initiation of this project, no study has been carried out to analyze the data regarding demographics, causes and degree of visual disability, and reasons for obtaining a UDID certificate. We designed this retrospective study to evaluate this data among visually disabled, registered individuals attending the ophthalmology outpatient department. We also wish to help the government in delivering the benefits of UDID cards to the target population by publicizing its details via mass media to improve awareness among the common public about the same.

## Materials and methods

The major objectives of this study were as follows: (i) to study demographic characteristics such as age, gender, and residence of UDID-certified visually disabled individuals; (ii) to study the primary condition causing visual disability and to classify visual disability in terms of low vision or blindness; and (iii) to ascertain the main reasons for seeking a visual disability certificate, such as getting government allowances, travel benefits, job reservations, and educational benefits.

This was a tertiary eye care center-based retrospective study and was approved by the institutional ethical committee. Patients with visual disabilities of 40% or above were included in the study. Written informed consent was taken from the patients. Records of individuals who came for obtaining UDID visual disability certificates were gathered from the Ophthalmology Outpatient Department, Government Medical College, Nagpur, Maharashtra. The study involved only urban-dwelling subjects of the city who came voluntarily for visual disability UDID certification. The study was conducted to analyze the demographic details of the patients such as age, gender, and residence, and also to classify the study individuals in terms of either low vision or blindness category as per the guidelines provided by the Government of India.

This retrospective study was conducted among patients belonging to urban Nagpur. The rural Nagpur area was designated to the Disability Board of the other Government Medical College in Nagpur, and hence patients belonging to rural Nagpur were not included in our study.

We included medical records and UDID portal data of 600 patients during the period from February 2019 to March 2022. All patients were first registered on the UDID online portal of the Government of India. Document verification was done by District Disability Rehabilitation Centre (DDRC) at our institute. Demographic characteristics like age, sex, and residence were documented. Detailed history and reasons for seeking a disability UDID certificate were noted. In all the subjects, a detailed ophthalmic examination was done, which included a recording of uncorrected and best-corrected visual acuity using the Snellen chart, anterior segment examination with slit-lamp biomicroscope, posterior segment evaluation with direct and indirect ophthalmoscope and 90 D lens, retinoscopy, automated refractometry (AR), tonometry, gonioscopy. optical coherence tomography (OCT), visual field charting using an Octopus Automated Perimeter, and ultrasonography (B-SCAN) of the eye as needed.

MRI plain and with the contrast of the brain and orbit, electroretinogram (ERG), visual evoked potential (VEP), and fundus fluorescein angiography (FFA) were done on patients as and when required. Subjective refraction was also done. If subjects were unable to read on the Snellen chart, visual acuity was measured by counting fingers by reducing distance sequentially from 6 meters to close to the face and further to the presence or absence of the perception of light. All the patients were examined by a concerned Disability Board member, who was a senior ophthalmologist. After the completion of the examination, the diagnosis was made. If the subject had multiple ocular diseases, the condition most likely responsible for visual disability was taken into account.

Patients with rejected UDID certificates, e.g., one-eyed patients, were excluded from the study. If the patients were found to have associated treatable diseases, they were first treated for the same and then included in the study accordingly. The percentage and category of visual disability were assessed according to the guidelines provided in the Gazette of India published on January 5, 2018, by the Ministry of Social Justice and Empowerment, India, based on best-corrected visual acuity and visual fields (Table 1) [4].

**Table 1:** Visual disability categorization* *Based on the most recent guidelines by the Gazette of India extraordinary part II section 3 published on January 5, 2018, by the Ministry of Social Justice and Empowerment, Government of India HMCF: hand movements close to the face

Better-eye best-corrected visual acuity	Worse-eye best-corrected visual acuity	Percentage of impairment	Disability category
6/6 to 6/18	6/6 to 6/18	0	0
	6/24 to 6/60	10	0
	Less than 6/60 to 3/60	20	I
	Less than 3/60 to "no light perception"	30	II (one-eyed person)
6/24 to 6/60 or visual field less than 40 to 20 degrees around the center of fixation or hemianopia involving the macula	6/24 to 6/60	40	III a (low vision)
	Less than 6/60 to 3/60	50	III b (low vision)
	Less than 3/60 to "no light perception"	60	III c (low vision)
Less than 6/60 to 3/60 or visual field less than 20 to 10 degrees around the center of fixation	Less than 6/60 to 3/60	70	III d (low vision)
	Less than 3/60 to "no light perception"	80	III e (low vision)
Less than 3/60 to 1/60 or visual field less than 10 degrees around the center of fixation	Less than 3/60 to "no light perception"	90	IV a (blindness)
Only HMCF or only light perception or "no light perception"	Only HMCF or only light perception or "no light perception"	100	IV b (blindness)

Data were entered into a Microsoft Excel spreadsheet and the final analysis was done with SPSS Statistics version 25.0 (IBM Corp., Armonk, NY). The main variables of our study were age, gender, the primary cause of disability, the percentage and category of disability, and reasons for seeking a visual disability certificate. Results were expressed in terms of mean, median, and range.

## Results

The age range in our cohort was 1-80 years, with the maximum number of individuals (n=214, 35.67%) falling under the age group of 11-30 years. The mean age was 35.56 ±18.6 years and the median age was 33.5 years (Table 2).

**Table 2:** Distribution of study subjects by age SD: standard deviation

Distribution of age (years)		
Mean ±SD	35.56 ±18.6	
Median (25th-75th percentile)	33.5 (20-50)	
Range	1-80	
Age group	Number of study subjects	Percentage (%)
1-10 years	52	8.67
11-20 years	100	16.67
21-30 years	114	19.00
31-40 years	89	14.83
41-50 years	101	16.83
51-60 years	79	13.17
61-70 years	51	8.50
71-80 years	14	2.33
1-10 years	52	8.67
11-20 years	100	16.67
21-30 years	114	19.00
31-40 years	89	14.83
41-50 years	101	16.83
51-60 years	79	13.17
61-70 years	51	8.50
71-80 years	14	2.33

**Figure 1:**
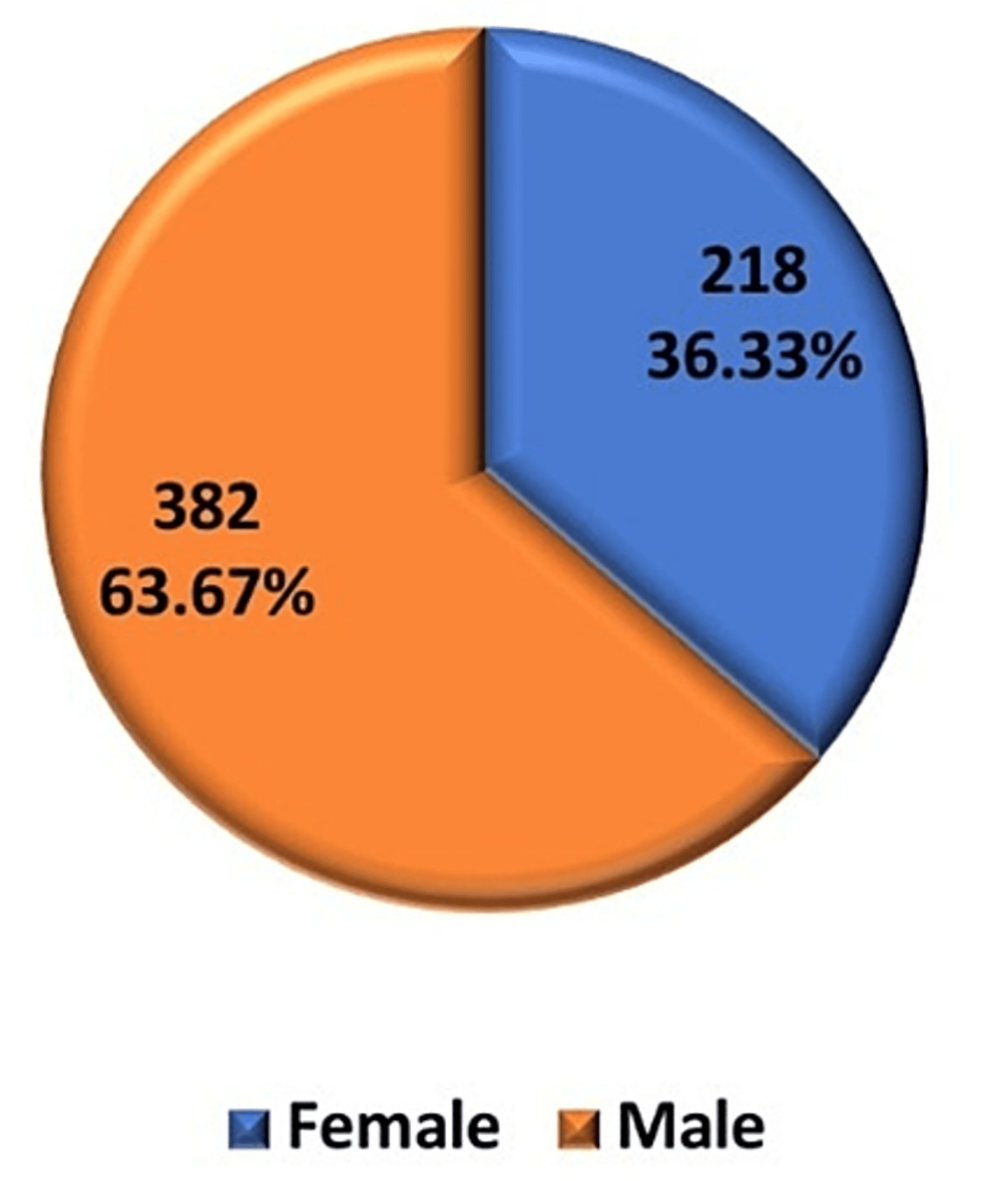
Gender distribution of subjects included in the study

Of the total 600 patients, 382 (63.67%) were males and 218 (36.33%) were females. The male-to-female ratio was 1.75:1, indicating a male preponderance. This can be attributed to the fact that males work more outdoors and hence they are more in need of the benefits offered by UDID cards as compared to females (Figure 1).

**Figure 2:**
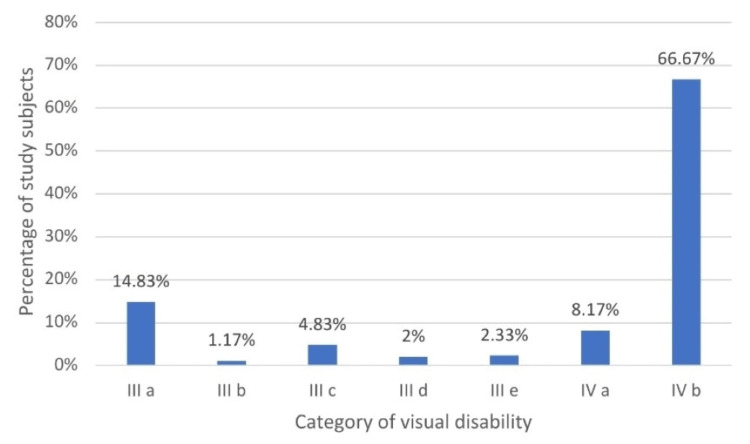
Distribution of study subjects according to the visual disability category

Of the total patients studied, 449 (74.84%) were categorized as blind (visual disability grade IV a, i.e., 90% and grade IV b, i.e., 100%) and 151 (25.16%) patients were under the low vision category (visual disability grade III a, i.e., 40% to grade III e, i.e., 80%) (Figure 2).

 Among all the retinal diseases, retinitis pigmentosa was the most common cause of visual disability (n=130, 21.67%) in the patients (Table 3).

**Table 3:** Detailed distribution of causes of visual disability in study subjects PHPV: persistent hyperplastic primary vitreous; FEVR: familial exudative vitreoretinopathy

Ocular diseases	Number of study subjects	Percentage (%)
Retinitis pigmentosa	130	21.67
Congenital anomalies	85	14.17
Optic atrophy	83	13.83
Amblyopia	47	7.83
Phthisis bulbi	42	7.00
Leukoma-grade corneal opacity	35	5.83
Macular diseases	33	5.50
High degenerative myopia	29	4.83
Glaucomatous optic atrophy	25	4.17
Anterior staphyloma	21	3.50
Ocular albinism	12	2.00
Failed retinal detachment surgery in both eyes	10	1.67
Chronic iridocyclitis	9	1.50
Retinopathy of prematurity	8	1.33
Corneal diseases (advanced keratoconus and failed keratoplasty)	6	1.00
Stevens-Johnson syndrome	5	0.83
Eviscerated eye	5	0.83
Complicated cataract	4	0.67
Chorioretinitis	3	0.50
Congenital glaucoma with buphthalmos	2	0.33
Cortical blindness	2	0.33
Rod cone dystrophy	2	0.33
Rare congenital retinal conditions (PHPV + FEVR)	2	0.33
Total	600	100.00

**Figure 3:**
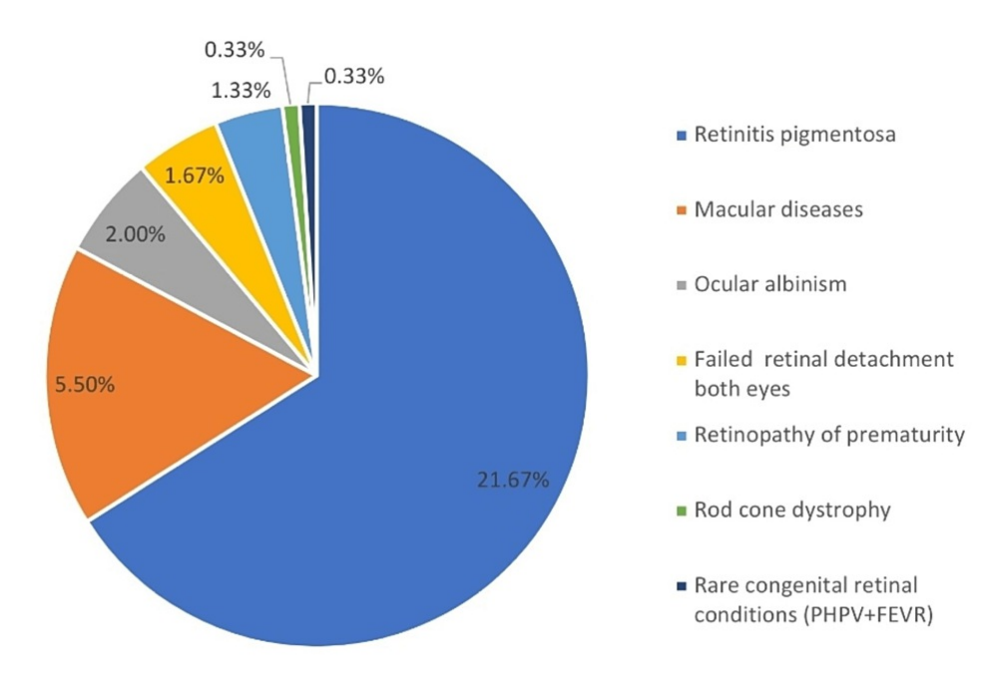
Distribution of retinal disorders among study subjects PHPV: persistent hyperplastic primary vitreous; FEVR: familial exudative vitreoretinopathy

Apart from retinitis pigmentosa, other retinal diseases found in the subjects were macular diseases, diabetic retinopathy, ocular albinism, high degenerative myopia, retinopathy of prematurity, failed retinal detachment surgeries, rod-cone dystrophy, chorioretinitis and rare congenital retinal disorders like familial exudative vitreoretinopathy (FEVR) and persistent hyperplastic primary vitreous (PHPV). Retinal diseases were the major cause of visual disability among the applicants, accounting for 229 (38.17%) patients (Figure 3).

Congenital anomalies comprising microcornea with microphthalmos, anophthalmos, sclerocornea, megalocornea, retinochoroidal colobomas, and aniridia accounted for a total of 85 (14.17%) patients (Table 3). Visual disability due to optic atrophy was found in 83 (13.83%) patients. Causes of optic atrophy included all types of primary and secondary optic atrophies, Leber’s congenital hereditary optic atrophy, Crouzon syndrome, and lasered proliferative diabetic retinopathy with consecutive optic atrophy (Table 3). Visual disability caused by amblyopia was seen in 47 (7.83%) patients. Causes of amblyopia were uncorrected refractive errors, anisometropia, nystagmus, congenital cataract, congenital ptosis, and strabismus (Table 3).

Phthisis bulbi was found in 42 (7%) patients. Causes of phthisis bulbi were trauma to the eye and corneal ulcers. Other causes of visual disability were leucoma grade corneal opacity (5.83%), glaucomatous optic atrophy (4.17%), anterior staphyloma (3.50%), chronic iridocyclitis (1.50%), corneal diseases (1%), Stevens-Johnson syndrome (0.83%), eviscerated eye (0.83%), complicated cataract (0.67%), congenital glaucoma (0.33%), and cortical blindness (0.33%) (Table 3).

**Figure 4:**
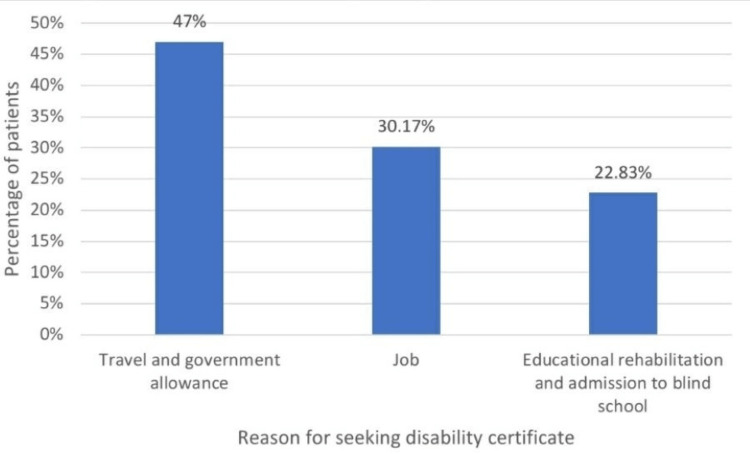
Distribution of reasons for seeking visual disability certificate among the study subjects

The reasons for seeking a visual disability certificate were as follows: getting government allowances and travel concessions in 282 patients (47%), getting jobs in the reserved category for persons with a disability in 181 patients (30.17%), and for educational benefits in 137 patients (22.83%). Many patients had multiple reasons for seeking the certificate, but only the main reason was taken into consideration for the purpose of this study (Figure 4).

## Discussion

UDID registration in India is voluntary and is done at the institute level: either the District Hospital or the Government Medical College in that area; individuals with any disability seeking governmental benefits may apply for UDID registration. Visual impairment is an important public health problem, especially in developing countries like India, as it impairs the quality of life and limits career choices and job opportunities, thereby constituting a socioeconomic burden on society [5,6].

In our study, a total of 600 patients who were assigned UDID visual disability certificates on the basis of permanent and non-treatable blindness/low vision were studied retrospectively. The data were collected from the UDID portal and medical records at the DDRC office.

In our study, the maximum number of individuals (n=214, 35.67%) were in the age group of 11-30 years. The majority of the study population was of younger age. This highlights the fact that younger people are more in need of benefits like educational help, reservation in jobs, financial assistance, tax benefits, concessions in various fields, and travel-related assistance. These findings are similar to those in the studies by Ghosh et al., Farooq et al., Khan et al., and Sadananda et al. [7-10]. In contrast, the UDID certification was very low in children below 10 years and elderly individuals above 70 years of age. This may be due to the lack of awareness about the online UDID registration portal, reduced need for certification, and higher dependency on other family members.

Of note, 63.67% of the subjects were males, and 36.33% were females, showing a male predominance in the study population. Similar gender bias was seen in other Indian studies, as shown in Table 4. This can be attributed to the fact that males work more outdoors and hence are more in need of the benefits than females. Females face inadequate access to healthcare facilities because of social barriers, low levels of literacy, higher dependency, and social discrimination [8].

**Table 4:** Comparison of our study parameters with similar previous studies

Study parameters							
	Our study	Ghosh et al. [7]	Khan et al. [8]	Farooq et al. [9]	Sadananda et al. [10]	Kareemsab et al. [11]	Joshi [12]
Sample size	600 patients (1200 eyes)	155 patients	553 patients	350 patients	551 patients	272 patients	279 patients
Duration	February 2019-March 2022	-	July 2018-July 2019	October 2017-October 2018	July 2019-March 2020	February 2009-August 2009	January 2008-December 2008
Population type	Urban	Rural	-	Urban and rural	Urban	Urban and rural	Urban and rural
Study design	Retrospective observational study	Cross-sectional study	Retrospective analysis	Cross-sectional study	Cross-sectional study	Prospective cross-sectional study	Retrospective analysis
Mean age	35.56 ±18.6 years	29.5 years	-	33.31 ±18.46 years	-	52.18 years	35.28 ±21.63 years
The most common age group with disability certificates	11-30 years	11-20 years	16-45 years	16-30 years	30-40 years	40-65 years	-
Male-to-female ratio	1.75:1	2.04:1	2.1:1	1.9:1	2.01:1	1.12:1	1.3:1
The most common cause of blindness	Retinitis pigmentosa	Phthisis bulbi	Phthisis bulbi	Corneal opacity	Retinitis pigmentosa	Congenital anomalies	Retinitis pigmentosa
Percentage of patients with 100% visual disability	66.67%	84.52%	19.89%	46.57%	95% with 90-100% blindness	55.15%	-
Cause of seeking a certificate	Travel and allowances	-	-	Financial and transport	-	-	Travel and educational purposes

All the patients enrolled in our study were from urban areas. India being a developing country, our urban population has higher rates of blindness compared to those in other Asian countries, which can be attributed to differences in socioeconomic conditions, quality of healthcare facilities, and access to eye care. Interpersonal communication can be a key tool for raising mass awareness among the target population regarding disability certification and its benefits. Emphasis should be given to comprehensive eye examinations by a healthcare professional even at the village level [8].

In our study, 400 (66.67%) patients were found to be 100% blind, i.e., category IV b. Similar results were found in studies by Ghosh et al., Kareemsab et al., and Sadananda et al. [7,10,11]; 49 (8.17%) patients were 90% blind, i.e., category IV a; 89 (14.83%) patients had 40% visual disability, i.e., category IIIa; and 62 (18.5%) patients had visual disability between 50% and 80%, i.e., category III b to category III e.

Our study found that retinal diseases (n=229, 48.50%) including retinitis pigmentosa (n=30, 21.67%) were the most common cause of visual disability. Other causes of visual disability were congenital anomalies (14.17%), optic atrophy (13.83%), amblyopia (7.83%), phthisis bulbi (7%), bilateral leucoma grade corneal opacities (5.83%), and macular diseases (5.50%). As retinitis pigmentosa was the major cause of blindness among the young population, avoidance of consanguineous marriages and provision of genetic counseling would play a vital role in preventing blindness. Screening of the target population will help in their early rehabilitation. Similar findings were found in studies done by Joshi and by Sadananda et al. [10,12].

Effective health policies are generally based on sound and reliable information about the disorder, and, therefore, well-designed epidemiological studies are needed to obtain reliable information about blindness in India. A well-planned investment in various aspects of blindness could lead to long-term savings in India’s resources by decreasing the socioeconomic burden of blindness [13].

At our institute, all one-eyed patients having <30% blindness in both eyes were rejected disability UDID certificates as they did not fit the criteria of disability certification by the Gazette of India, extraordinary. These one-eyed persons neither had normal vision in both eyes nor were they considered as having a visual disability. Hence, they cannot get jobs and other benefits of UDID certification. We believe that the governing authorities should make necessary changes in the UDID disability criteria to make these one-eyed persons eligible for UDID cards [8].

The rehabilitation of people with visual impairment has been relatively ignored in India due to the lack of postgraduate training in the field of low vision rehabilitation, the general perception being that it is time-consuming and generally unsuccessful, which is compounded by the very poor availability of locally made low vision aids. Laptop computers are excellent aids for the visually impaired. The rapid development of computer technology for the visually impaired is very exciting [14]. Hence, we recommend that low vision aids should be made available at all government institutes for UDID cardholders free of cost to rehabilitate them individually and socially.

Limitations

As this was a hospital-based study involving visually disabled individuals from urban areas who voluntarily registered for UDID certificates, it does not reflect the true prevalence of low vision and blindness in the general population. Data on the true prevalence can only be obtained by door-to-door surveys and screening programs at the grassroots level. Another limitation of our study was that we could not study certain demographic details such as the educational status and occupation of the study subjects.

## Conclusions

In our study, the most common cause of visual disability was retinal diseases including retinitis pigmentosa followed by congenital anomalies, optic atrophy, amblyopia, and phthisis bulbi. The most common age group was 11-30 years with males being significantly more in number than females. Around 67% of patients had 100% visual disability/blindness. The most common reason for obtaining a UDID certificate was for getting financial help and travel benefits from the Government.

We believe that the findings of our study would be useful to Government agencies to plan strategies for the prevention of blindness in the general population and implement programs for the rehabilitation of blind persons. It would also help the Government to prioritize healthcare services in order to treat the diseases causing blindness at the earliest. Mass awareness campaigns and using social media to educate people about the UDID card and its benefits would certainly help increase the registration of visually disabled individuals for certification and their rehabilitation. Genetic counseling would help in the prevention and early rehabilitation of patients with retinitis pigmentosa.
